# Mechanistic insights into cadmium-related premature aging in *Drosophila* model

**DOI:** 10.3389/fnins.2025.1605687

**Published:** 2025-06-04

**Authors:** Maria Dolores De Donno, Ester Mercuri, Barbara Balena, Francesco Zangaro, Maria Pia Bozzetti, Valeria Specchia

**Affiliations:** Department of Biological and Environmental Sciences and Technologies, University of Salento, Lecce, Italy

**Keywords:** *Drosophila melanogaster*, cadmium, aging, neurodegeneration, cognitive impairments, LLPS

## Abstract

The intricate and multifaceted relationship between environmental pollutants, particularly heavy metals such as cadmium, and human health has been extensively documented, with a significant focus on their neurotoxic effects. Notably, the connection between cadmium exposure and Alzheimer’s disease is becoming increasingly evident, prompting a deeper investigation into the underlying mechanisms at play. Despite the growing body of evidence linking cadmium to neurodegeneration and although harmful molecular activities of cadmium in cells have been demonstrated, the precise molecular mechanism induced by this toxic metal within neuronal cells remains largely enigmatic. This study aims to shed light on these mechanistic processes by utilizing *Drosophila melanogaster,* a widely recognized model organism in neurogenetics, as our experimental framework. Through a carefully designed approach, we simulated chronic exposure to cadmium, which allowed us to observe the resulting effects on the flies over time. Our findings revealed that chronic cadmium exposure led to premature aging in flies, characterized by neurodegeneration and an exacerbation of complex neurological phenotypes. Notably, these included significant impairments in learning and memory, which are critical cognitive functions often compromised in neurodegenerative conditions. With the aim of exploring the mechanistic underpinnings of these observations, we determined that cadmium impairs RNP formation and could disrupt the delicate process of liquid–liquid phase separation within neuronal cells. This disruption appears to play a pivotal role in initiating the cascade of events that contribute to neurodegeneration. Liquid–liquid phase separation is essential for the proper organization of cellular components and the maintenance of neuronal health; thus, cadmium’s interference in this process may provide a crucial insight into its neurotoxic effects.

## 1 Introduction

### 1.1 Environmental contaminants and human health: a focus on cadmium

The relationship between environment and human health is established and multifaceted. Environmental factors such as air and water quality, chemical exposures, and climate change have profound impacts on human health. Poor environmental conditions can lead to a variety of health issues, including neurodegenerative diseases, respiratory and cardiovascular problems, weakened immune systems (Shetty et al., 2023). Chemicals found in the environment, such as pesticides and heavy metals, can have serious effects on human health. Among them, cadmium is a toxic heavy metal commonly found in the environment, particularly in industrial areas and as a contaminant in food products. It has garnered significant attention due to its potential neurotoxic effects on humans (Genchi et al., 2020). Cadmium exposure has also been associated with neurodegenerative diseases such as Alzheimer’s disease (AD), Parkinson’s disease (PD), and amyotrophic lateral sclerosis (ALS) (Min and Min, 2016; Viaene et al., 2000; Raj et al., 2021).

The molecular mechanisms by which cadmium affects neurons are complex and not completely understood. Understanding these molecular mechanisms, specifically the pathways involved in the early timing of exposure to cadmium, is crucial for developing strategies to mitigate its effects on human health and for establishing regulatory policies to limit exposure to this harmful substance. This aspect is even more important for neurodegenerative and age-related diseases that affect a very large number of people (Hou et al., 2019).

### 1.2 The importance of model organisms to study the effects of cadmium: a focus on *Drosophila*

In this context, animal model organisms play a crucial role in understanding the molecular pathways involved in physiological aging and neurodegenerative diseases, and accordingly in determining how neurons respond to the uptake of chemical pollutants (Ali et al., 2021; Yang et al., 2022). Thanks to its short lifespan, *Drosophila melanogaster* serves as an excellent model for studying the molecular pathways underlying the physiological processes of aging, as well as human age-related and neurodegenerative diseases. Lifespan measurement is a fundamental method for assessing the effects of both genetic and non-genetic factors involved in aging. In addition, in *Drosophila melanogaster*, age-related decline is observed in neural functions, including learning and memory loss, and diminished olfactory capabilities (Iliadi and Boulianne, 2010; Kanellopoulos et al., 2012; Haddadi et al., 2014; Nitta and Sugie, 2022). At the same time, *Drosophila*’s relatively simple nervous system, combined with sophisticated genetic tools, allows researchers to dissect the molecular and cellular mechanisms underlying neurodevelopment, neurodegeneration and neurodegenerative diseases (Lu and Vogel, 2009; Kosmidis et al., 2011; Bozzetti et al., 2015), opening the importance to use this model to study the effects of cadmium on neurodegeneration.

Regarding the studies conducted so far with cadmium in *Drosophila*, a recent study has focused primarily on fertility issues. A cadmium dose-dependent decline in fecundity in the parental generation was observed, linked to oxidative stress and disrupted ion transport affecting follicle development. Transcriptome analysis identified early molecular responses involving the Wnt and mTOR signaling pathways. Notably, detrimental effects persisted for two to three generations after cadmium removal, with epigenetic modulation of Dnmt2 influencing fecundity across generations (Pan et al., 2025). Another study investigated the impact of cadmium exposure on the growth, development, activity, sleep, and ferroptosis in *Drosophila*. Exposure to cadmium prolonged development, reduced pupation and eclosion rates, decreased body weight and size, and increased mortality. It also altered behavior, decreasing activity and increasing sleep (Hu et al., 2023).

### 1.3 Stress sensor pathways activated by cadmium: an overview

The Stress-Activated Protein Kinase (SAPK) pathway, a component of the broader MAPK family, is activated in response to various stress stimuli and acts as key mediators in cellular responses to stress and damage. The SAPK pathway is highly conserved evolutionarily, highlighting its importance in cellular stress responses across species (Kobayashi et al., 2001; Nishina et al., 2004; Du et al., 2025). This pathway plays a significant role in regulating processes such as apoptosis and inflammation (Kyriakis and Avruch, 2001; Hu et al., 2025).

SAPK kinase is an upstream activator of cJUNK and p38 MAP kinase in the signaling cascade that transmits stress signals within the cell. Activated p38 proceeds to phosphorylate a variety of downstream substrates, including transcription factors, enzymes, and other proteins, thereby mediating cellular responses to stress, such as inflammation and apoptosis.

In *Drosophila melanogaster*, this pathway has been studied to understand its role in stress response and organismal survival. The activation of the *Drosophila* SAPK pathway modulates stress tolerance and influences lifespan under challenging conditions (Kobayashi et al., 2001; Sakakibara et al., 2023). Cadmium exposure activates the SAPK pathways in mammalian cells, contributing to apoptosis and inflammatory responses (Jung et al., 2008). In *Saccharomyces cerevisiae*, cadmium exposure triggers stress responses involving MAPK pathways, particularly the Hog1 pathway, which is analogous to the p38 pathway (Du et al., 2025).

In the mouse model, exposure to cadmium activates SAPK pathway and exacerbates neurodegeneration (Ali et al., 2021).

Overall, SAPK pathway precise regulation is vital for maintaining cellular homeostasis and preventing pathological conditions such as neurodegeneration (Ferrer et al., 2001; Ali et al., 2021).

### 1.4 The role of ribonucleoprotein aggregates and liquid–liquid phase separation process in neurodegeneration

A clear link has been established between accumulation of aberrant ribonucleoprotein (RNP) aggregates and progression of aging-related neurodegenerative diseases. In a healthy nervous system, dynamic RNP granules have been observed, exhibiting both unique and overlapping compositions. In neurodegenerative diseases like Frontotemporal Degeneration (FTD) and Amyotrophic Lateral Sclerosis (ALS), the accumulation of static RNP aggregates with abnormal composition appear. These pathological aggregates are thought to sequester RNA-binding proteins (RBPs), potentially contributing to disease progression (Pushpalatha et al., 2022).

In *Drosophila*, upon physiological aging, cytoplasmic RNP components progressively condense into large yet dynamic granules in neurons. Age-related changes in RNP granules are linked to the translational repression of mRNAs recruited to these structures, suggesting a suppressive role for neuronal RNP granules (Pushpalatha et al., 2022).

RNA-binding proteins and RNAs dynamically condense into RNP granules through the liquid–liquid phase separation (LLPS) process (Shin and Brangwynne, 2017). Numerous studies highlight that LLPS is a driven process that occurs spontaneously due to changes in molecules concentration and intermolecular interactions (Falahati and Haji-Akbari, 2019). LLPS is critical for organizing cellular components and facilitating efficient biochemical reactions (Gomes and Shorter, 2019). Under normal conditions, LLPS allows these granules to remain highly dynamic, with continuous exchange of their molecular components. However, when LLPS is dysregulated, it can lead to the formation of aberrant, less dynamic aggregates (Zbinden et al., 2020; Wang et al., 2021).

Furthermore, LLPS plays a pivotal role in the misfolding and aggregation of proteins, particularly tau and amyloid-beta, in Alzheimer’s disease (AD). Dysregulated LLPS contributes to the pathological hallmarks of AD by promoting the transition from liquid-like condensates to insoluble aggregates (Wegmann et al., 2018; Fu et al., 2024).

In this research, we used the *Drosophila* model to investigate the molecular effects of chronic exposure to cadmium on the brain unveiling an intriguing molecular mechanism that may be based on the liquid–liquid phase separation process.

## 2 Methods

### 2.1 Flies stocks

In this study the following strains of *Drosophila* are used: *Oregon-R* wild type strain as control; *moody-GAL4* strain (BDSC 90883) crossed with *UAS-mCD8: GFP* strain (BDSC 64305); *Imp-EGFP* strain (BDSC 60237). Flies are reared in plastic vials containing standard cornmeal agar medium at 25°C and exposed to a 12:12 h light–dark cycle.

### 2.2 Treatment with cadmium in the diet

To test the cadmium toxicity on *Drosophila melanogaster*, we prepared three cadmium chloride solutions at concentration of: 0.05 mM, 0.1 mM and 0.5 mM. To administrate the cadmium to the flies, we inserted a paper impregnated with 2 ml of each cadmium concentration into the culture tubes.

We counted the number of dead flies after 15 days and we obtained 50% mortality at day 15 (LC₅₀) with the 0.1 mM concentration. We studied the effect of cadmium on flies putting the flies directly after their eclosion on the cadmium medium.

### 2.3 Measurement of lifespan in *Drosophila melanogaster*

100 flies (50 males and 50 females), control and treated with 0.1 mM cadmium concentration are used for lifespan measurement. During the experimental period, flies are transferred onto new vials containing fresh food every 2 days and the number of dead flies is recorded, until the last survivor is dead. The data are entered and analysed on Excel to create the survivorship curve.

### 2.4 Learning and memory assay or behavioral experiments

In our test we use a T-maze trial based on the Pavlov Studies (Tully and Quinn, 1985). T-maze system consists of two chambers, a dark one and a light one separated by a sliding plate. Physiologically, flies move from dark to light, but in the experiment, the light is associated with a negative stimulus, 0.1 M Quinine solution.

For the experiment flies positive for phototaxis are selected and used for the test. During the test, flies are left in the dark for 30″, to allow acclimatization. After this time, the light is turned on and the plate separating the two chambers is opened. The flies enter the light chamber and walk for about 30″ on the filter paper containing the quinine. The learning steps are repeated four times and the number of flies that go toward the light is counted. To analyze the memory, the flies are left for 5 h at 25°C, after that they are re-tested.

### 2.5 Total RNA extraction

Total RNA is extracted from adult heads (25 males and females flies for each extraction) using an RNAqueous™-Micro Total RNA Isolation Kit (Invitrogen, Waltham, MA, USA) and following the manufacturer’s protocol. To remove all the DNA in the preparation, samples are incubated with DNase I RNase free at 37°C for 20 min; after treatment, DNase is inactivated using the DNase Inactivation Reagent from the kit.

### 2.6 cDNA synthesis from total RNA

For first-strand cDNA synthesis, up to 5 micrograms of total RNA is used as a template for oligonucleotide dT(20) primed reverse transcription using the SuperScript III First-Strand Synthesis System (Invitrogen, Waltham, MA, USA) according to the manufacturer’s instructions.

### 2.7 Quantitative real-time PCR

Real-time PCR is performed with the StepOne Real-Time PCR (Applied Biosystems, Invitrogen, Waltham, MA, USA) system. Expression of genes is determined by real-time PCR using PowerUp™ SYBR™ Green Master Mix (Applied Biosystems, Invitrogen, Waltham, MA, USA) according to the manufacturer’s protocol. For quantification of the transcripts, we used the 2DDct method (Livak and Schmittgen, 2001) with rp49 transcripts as control. All primers are listed in Supplementary Table S1.

### 2.8 Statistical analysis

For comparisons between two measurements, a two-tailed Student’s *t*-test is used to show statistical significance. The significance threshold is set as follows: *p < 0.05, **p < 0.01 and ***p < 0.001.

### 2.9 Immunostaining and confocal microscopy

Drosophila brains are dissected in phosphate-buffered saline (PBS) and fixed in 4% paraformaldehyde solution for 20 min, followed by three washes in PBS with 0.3% Triton X-100 (PBST) for 15 min. The tissues are blocked with 5% of Normal Goat Serum (NGS) for 60 min and incubated with primary antibody anti-GFP (rabbit polyclonal, NB600-308, Novus Biologicals, Littleton, CO, USA; 1:200) overnight at 4°C. The day after, tissues are washed three times with 0.3% PBST for 15 min and blocked with 5% NGS for 60 min. Tissues are incubated with 1:500 FITC-conjugated anti-rabbit-IgG antibody (Jackson) for 120 min and then are washed three times with 0,3% PBST for 15 min. At the end the tissues are mounted on a slide with SlowFade™ Diamond Antifade Mountant with DAPI (Thermofisher Scientific) according to the manufacturer’s instructions. Samples are examined and images are captured using a laser-scanning confocal microscope (Zeiss LSM 700).

### 2.10 Apoptosis detection by TUNEL assay

*Drosophila melanogaster* dissected brains are used as targets for TUNEL reaction. The TUNEL assay is performed using the TUNEL Assay Kit (Fluorescence, 488 nm) (Cell Signaling Technology) and following the manufacturer’s protocol. Samples are examined and images are captured using a laser-scanning confocal microscope (Zeiss LSM 700).

## 3 Results

### 3.1 Learning and memory impairment in cadmium-treated flies

**Figure 1:**
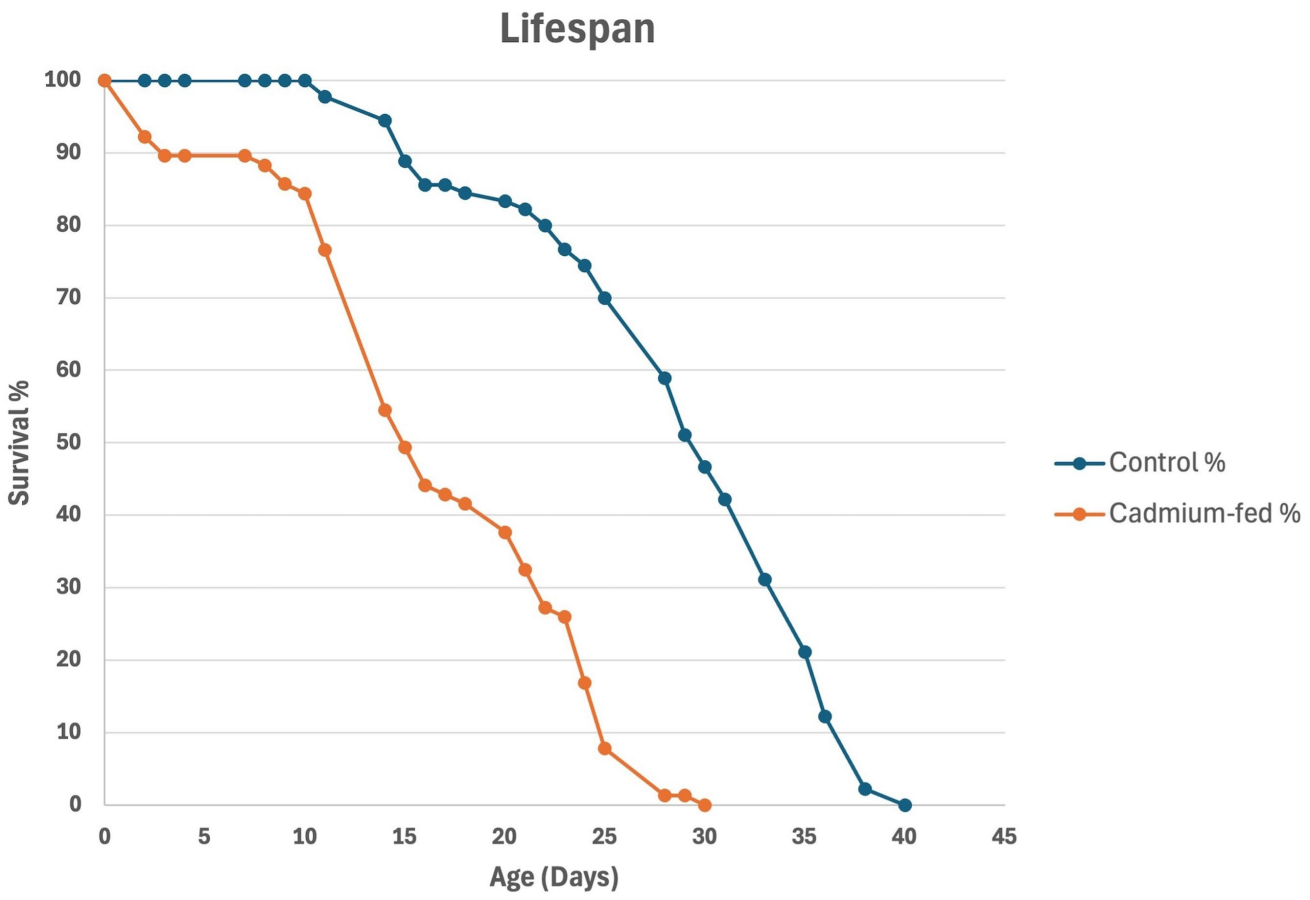
Survival curve of control strain (blue line) and cadmium-treated flies (orange line). In the control group, a gradual population decline is observed in 40 days. In contrast, the cadmium-treated group exhibited a more rapid decline in survival: after 15 days, the cadmium-treated group was halved and by day 30, all individuals in this group died, indicating a drastic reduction in lifespan compared to the control group.

To determine if *Drosophila* is an effective model for studying the relationship between cadmium and neurodegeneration, we examined whether exposure to this metal leads to the development of complex neurological phenotypes. First of all, we verified the toxicity of cadmium using three different concentrations of cadmium chloride, 0.5 mM, 0.1 mM, and 0.05 mM. For each concentration, we assessed the effects by monitoring the survival rate of flies. With the aim to simulate chronic cadmium exposure and evaluate its impact on neurodegeneration and aging, we selected the 0.1 mM concentration because it resulted in approximately 50% mortality at 15 days, allowing us to model chronic exposure while maintaining a sufficient number of surviving flies, although the lifespan has been found to be shortened compared to untreated control flies (Figure 1).

**Figure 2:**
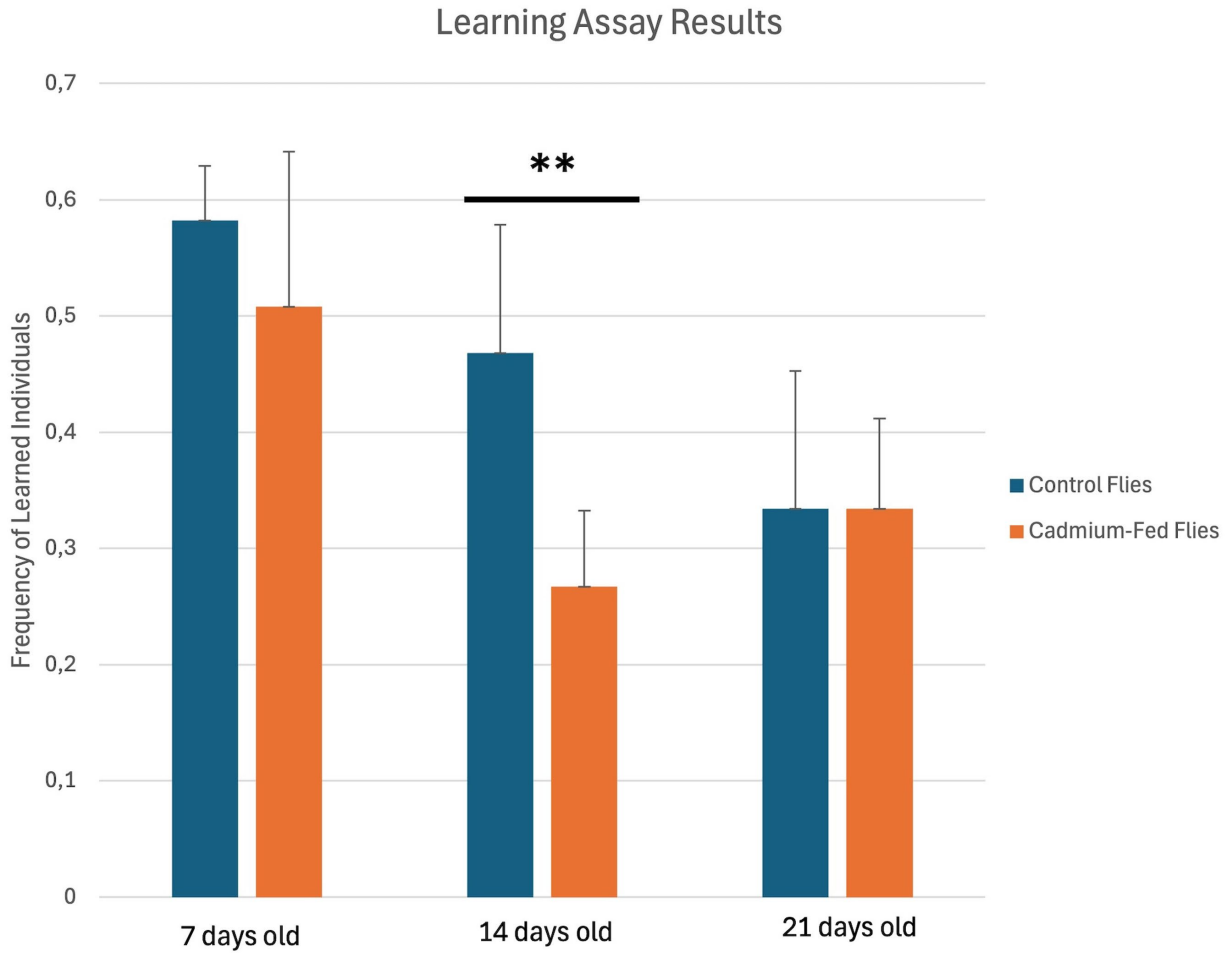
Learning Assay Results in individuals of *Drosophila melanogaster* control strain (blue) and cadmium-fed flies (orange). Specifically, for learning assays, values represent mean ± SD of *n* = 5 groups (total ~ 100 flies). For each group, flies positive to phototaxis were trained four times for the ability to associate light with the negative stimulus that inhibits phototaxis. For treated and control flies, the fractions of individuals positive (unlearned) and negative (learned) to phototaxis at the last training were calculated *p < 0.05, **p < 0.01, and ***p < 0.001 by Student’s *t*-test.

**Figure 3:**
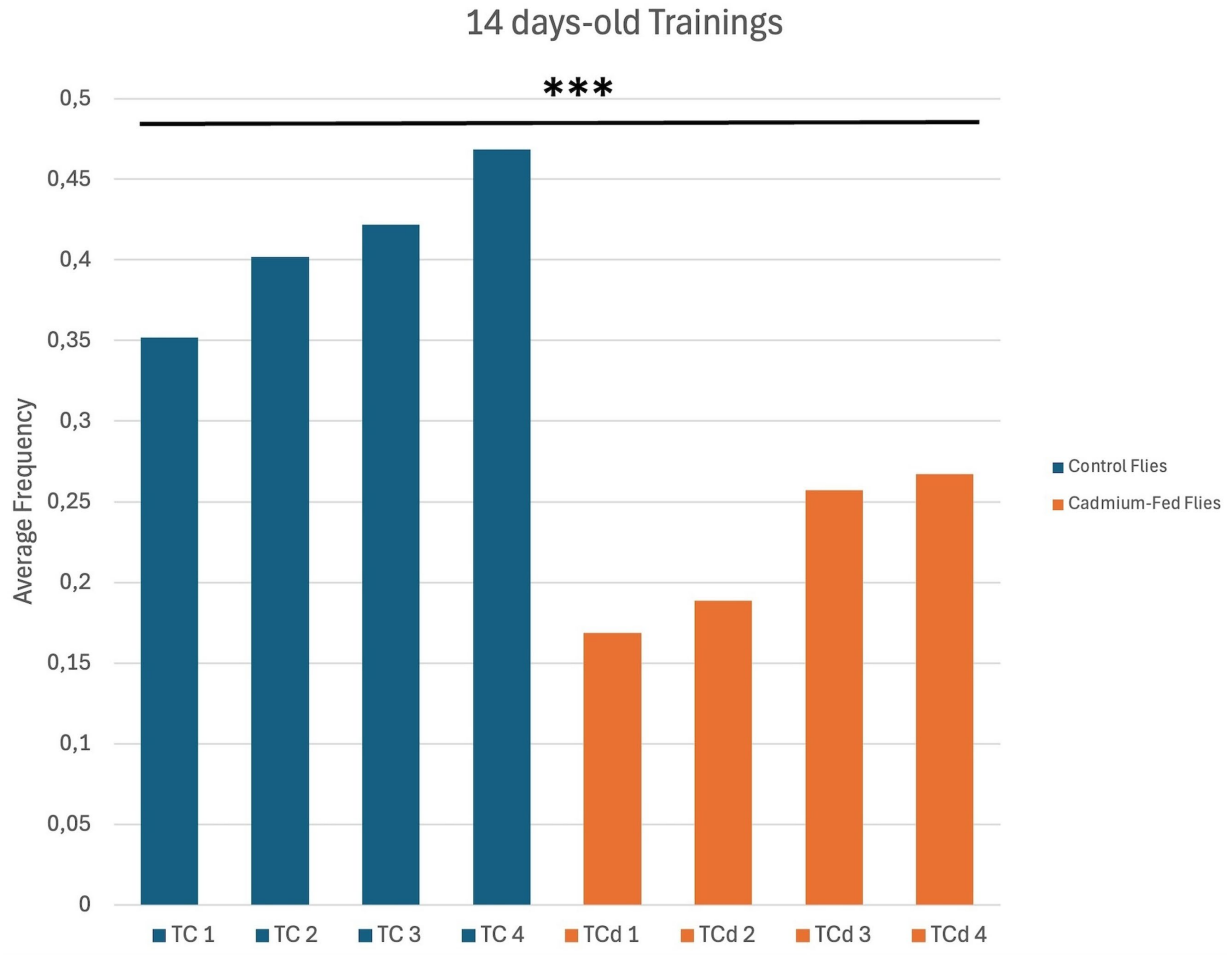
Average frequency of the 5 groups for each test [Test Control Flies (TC) 1–4; Test Cadmium-Fed Flies (TCd) 1–4]. For 14 days-old treated and untreated flies, the difference between fractions of individuals positive (unlearned) and negative (learned) to phototaxis were calculated for each test (1–4). ***p < 0.001 by ANOVA test which revealed a highly significant difference across the four training sessions in the treated groups compared to the control groups.

Wild type flies that were treated in their diet with cadmium 0.1 mM after their eclosion for 7, 14, and 21 days of age were assessed for their learning and memory capacity by using a taste aversion assay. Hundred flies, cadmium-treated and exhibiting positive phototaxis, were divided into five groups and tested. Hundred control flies, exhibiting positive phototaxis, were also divided into five groups for testing. For each group, flies were trained iteratively for a series of times (tests 1–4) to evaluate their ability to associate light with a negative stimulus represented by a quinine solution 0.1 M which has a bitter taste and that inhibits phototaxis. Specifically, 14 day-old cadmium-treated flies displayed cognitive defects compared to untreated normal 14 day-old flies, with a significant reduction in learning (Figures 2, 3).

**Figure 4:**
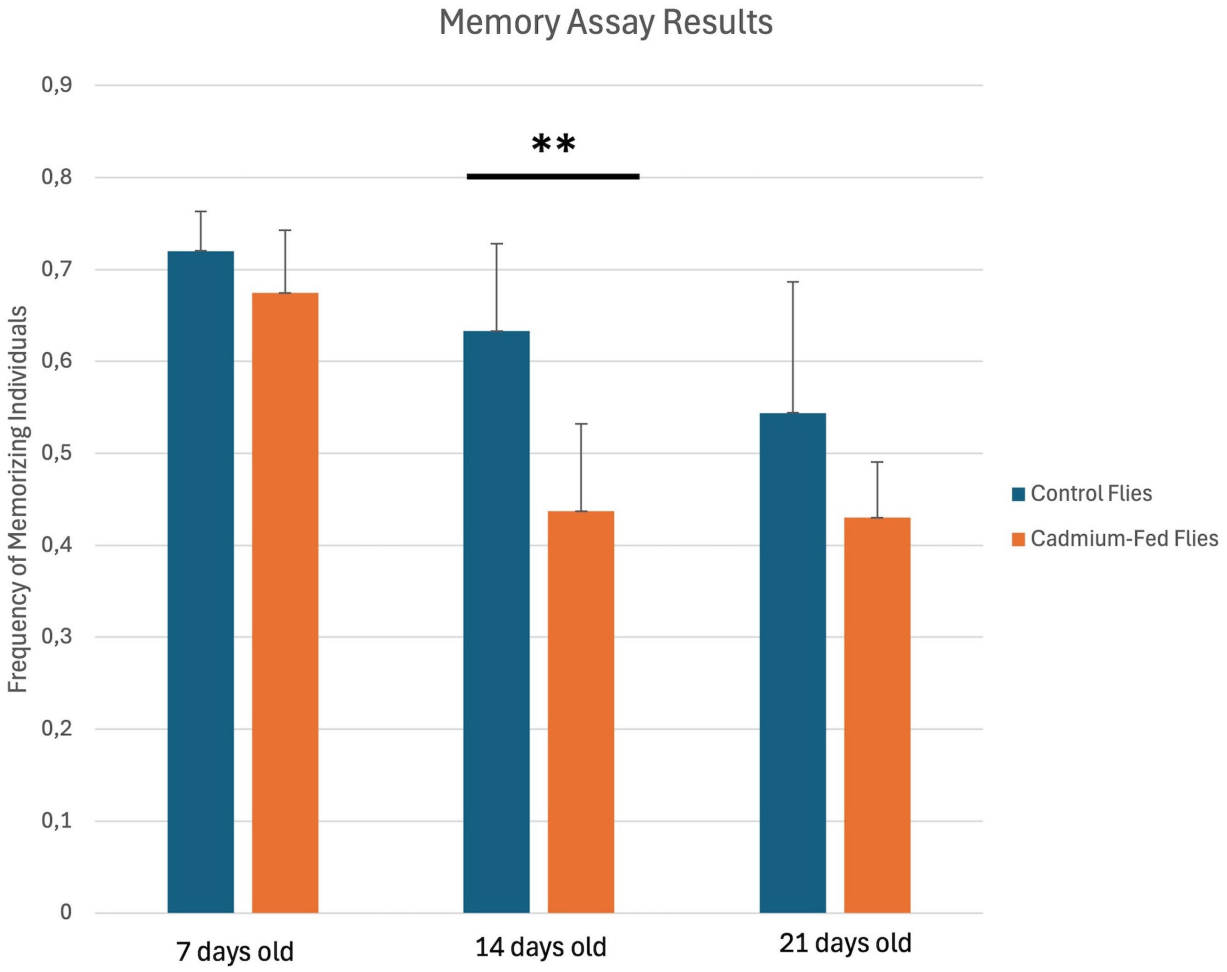
Memory Assay Results in individuals of *Drosophila melanogaster* control strain (blue), and cadmium-fed flies (orange). For short-term memory assays, values represent mean ± SD of an *n* = 5 groups. Single groups of trained flies were left for 5 h at 25°C and re-tested. The fractions of individuals positive (memory-defective) and negative (memorizing) to phototaxis were calculated *p < 0.05, **p < 0.01, and ***p < 0.001 by Student’s *t*-test.

In addition, memory abilities are significantly reduced in 14 day-old cadmium-treated flies compared to untreated normal 14 day-old flies (Figure 4). Therefore, cadmium appears to cause premature aging in flies because it has been well established that aging significantly impacts cognitive processes, leading to declines in both learning abilities and memory retention (Kandel, 2001; Murman, 2015).

### 3.2 Activation of molecular markers of stress in the brains of cadmium-treated flies

**Figure 5:**
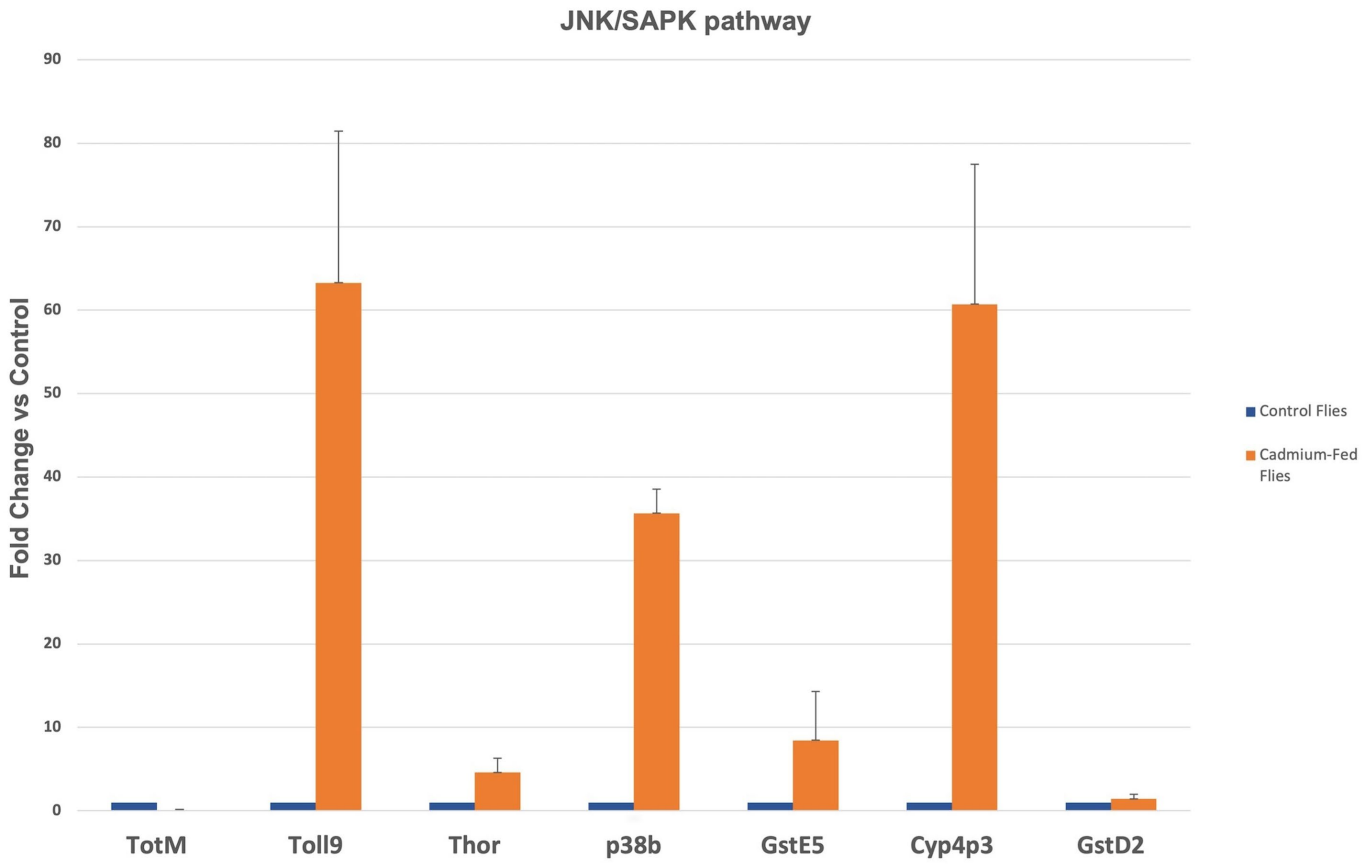
The mRNA expression levels of SAPK pathway genes in 14 days-old cadmium-treated fly heads vs. control.

To investigate if the stress sensor pathways are activated after cadmium treatment, we examined the mRNA expression levels of genes downstream of SAPK, JNK and NF-kB, JNK, and SAPK pathways in heads of 14 day-old flies treated with cadmium after eclosion against age-matched control by qRT-PCR (Figure 5).

We obtained that the JNK/SAPK signaling pathway is strongly activated in cadmium stressed brains, especially Toll-9, p38b and Cyp4p3. The mRNA expression levels of Toll-related genes is elevated in treated fly heads, and the genes downstream of JNK (Thor) and SAPK (TotM, p38b, GstE5, Cyp4p3, GstD2) pathways are upregulated in treated fly heads. Overall, the results indicate that cadmium activated stress sensor and signaling pathways in the brains of *Drosophila.*

### 3.3 Cellular markers of neuronal death appear in cadmium-treated flies

**Figure 6:**
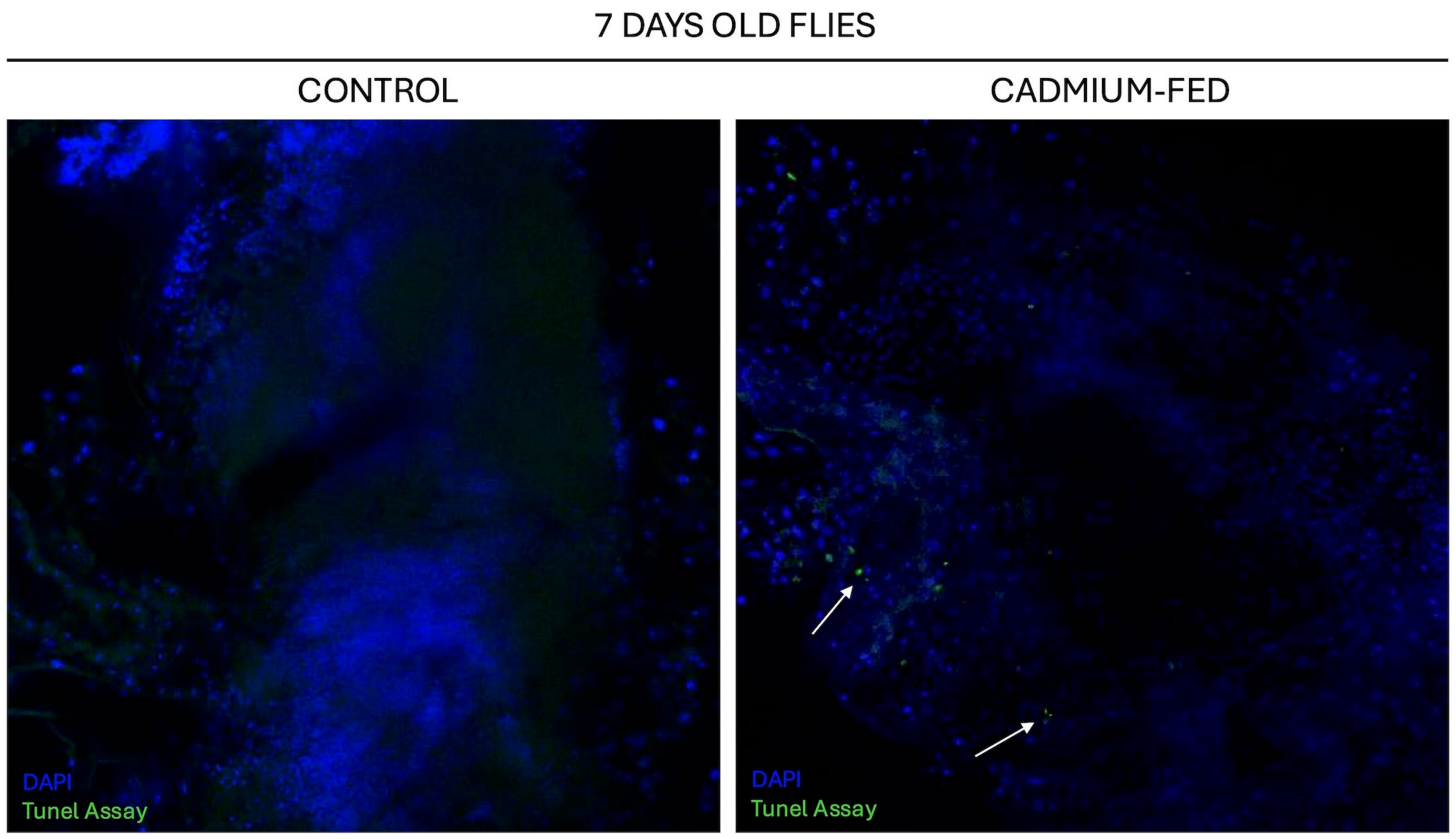
Confocal analysis of *Drosophila melanogaster* brain section of 7 days-old flies treated with cadmium **(right)** against control **(left)** using Tunel Assay Kit (green) and DAPI (blue). White arrows indicate Tunel positive nuclei. Magnification 40X.

**Figure 7:**
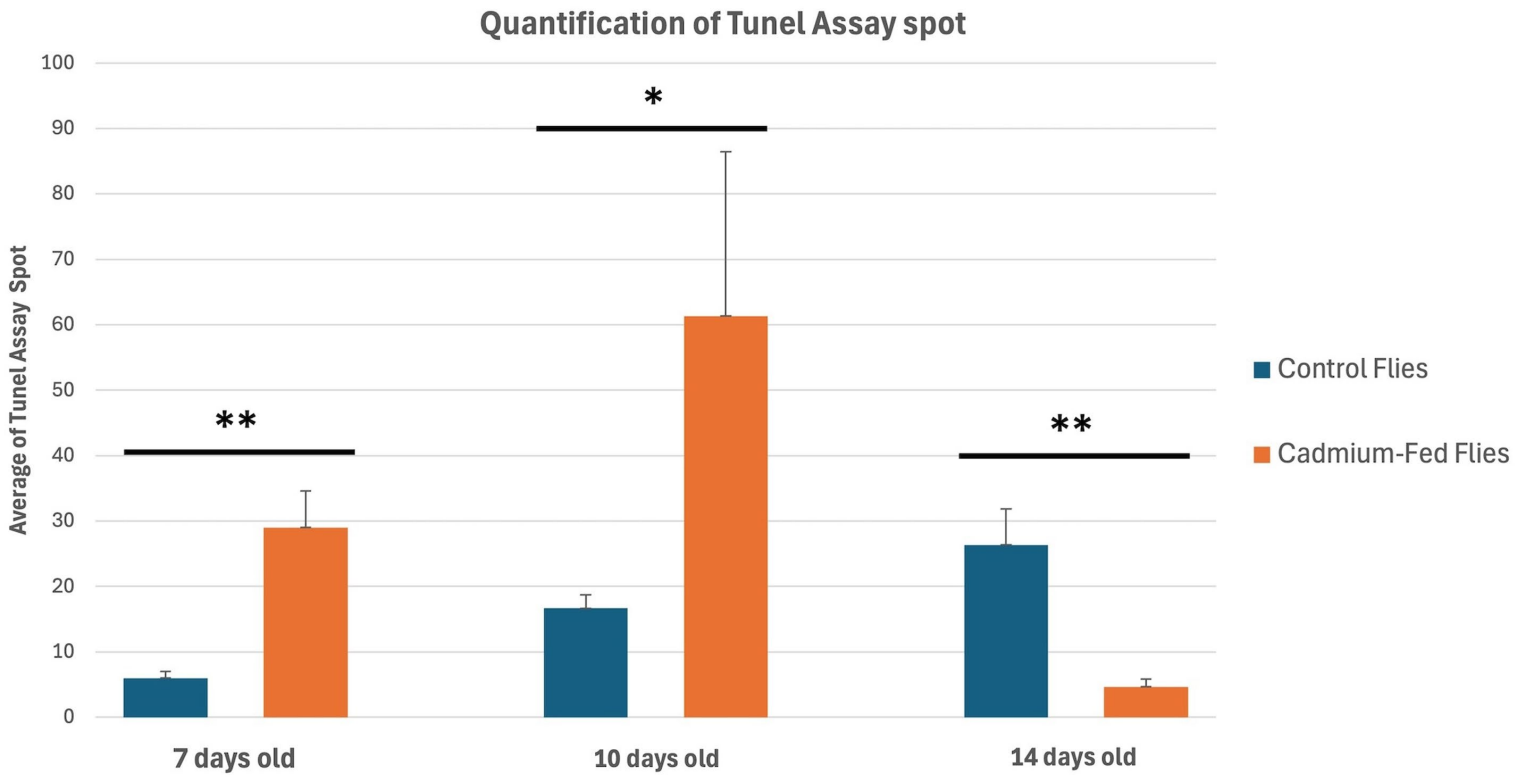
Quantification of Tunel Assay spot in *Drosophila melanogaster* brain. Mean ± SEM, *n* = 3; *p < 0.05, **p < 0.01, and ***p < 0.001 by Student’s *t*-test.

To determine the relationship between cognitive defects induced by cadmium exposure and neurodegeneration processes, we conducted a comparative analysis of brain tissue from individuals treated with cadmium at 7, 10, and 14 days of age against age-matched control groups. This analysis focused on identifying the presence of cell death markers, such as apoptosis and necrosis, in the brain tissue samples. We employed TUNEL staining to visualize and quantify cell death. Statistical analyses were carried out to evaluate the significance of differences observed between cadmium-treated groups and controls (Figures 6, 7).

In the control group, a gradual increase in TUNEL signals is observed over time; in contrast, in the cadmium-treated group, high TUNEL signals were already detected at 7 days and further increased at 10 days (Supplementary Figure S1). By 14 days, a complete decline in cell death signals is observed. In summary, cell death begins after 7 days in cadmium-treated flies, reaching its maximum at 10 days, which could be correlated with a subsequent decline in *Drosophila* behavioral abilities, as observed at 14 days through learning and memory tests, along with the activation of the JNK/SAPK pathway.

Our findings aimed to elucidate the extent to which cadmium exposure correlates with neurodegenerative changes and, consequently, cognitive impairments in the affected individuals. By correlating the level of cell death with cognitive performance metrics from behavioral assessments, we aimed to establish a clearer link between neurodegeneration processes and cognitive defects associated with cadmium treatment.

### 3.4 The blood–brain barrier maintains its integrity in the brains of cadmium-treated flies

**Figure 8:**
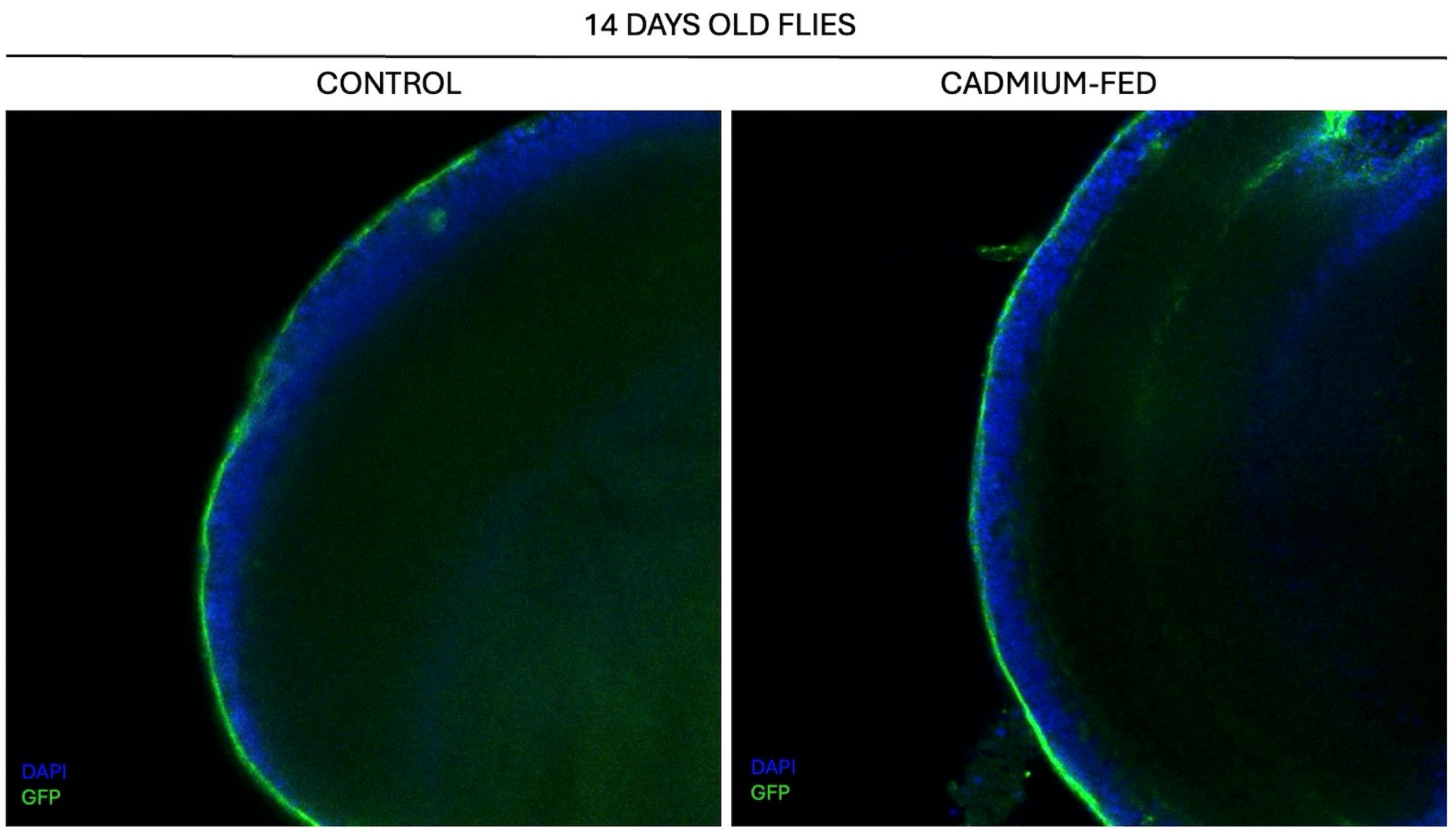
Confocal analysis of BBB in 14 days-old flies of control *moody-GAL4/UAS-mCD8: GFP* strain **(left)** and of cadmium-fed *moody-GAL4/UAS-mCD8: GFP* strain **(right)**. Tissue stained with GFP (green) and DAPI (blue). Magnification 40X.

Since cadmium can cause a variety of issues in *Drosophila melanogaster*, it is important to investigate the mechanisms by which this heavy metal acts within the nervous system, starting with its ability to reach the brain of *Drosophila* and affect its functions. We verified the integrity of the blood–brain barrier in *Drosophila melanogaster* to understand the role of cadmium, specifically whether it can alter the barrier’s integrity or if it simply crosses the barrier. We use the UAS-GAL4 system to generate a line capable of expressing GFP within the cells of the blood–brain barrier (BBB). The *moody-GAL4* driver line was crossed with the *UAS-mCD8: GFP* responder line, and the 14 days-old progeny brains are used for immunostaining experiments with an antibody anti-GFP (Novus, NB600-308) and with DAPI (Figure 8).

From this analysis, it appears that cadmium does not alter the integrity of the BBB, and it might cross it without any issues. It can enter the brain through transport mechanisms such as those used for other metal ions or by directly interacting with receptors and transporters in the BBB.

### 3.5 Neuronal RNP component prematurely condenses in cadmium-treated flies

To investigate the molecular mechanism triggered by the entry of cadmium into the neuron, we have tested the intriguing hypothesis of a role for cadmium in influencing the liquid–liquid phase separation process. Specifically, the liquid–liquid phase separation mechanism drives the formation of ribonucleoprotein (RNP) complexes in neurons. Additionally, it has been observed that pathological conditions, such as neurodegenerative diseases, can disrupt the normal phase separation of RNPs, leading to the formation of aggregates that may contribute to cellular dysfunction (Alberti et al., 2019).

**Figure 9:**
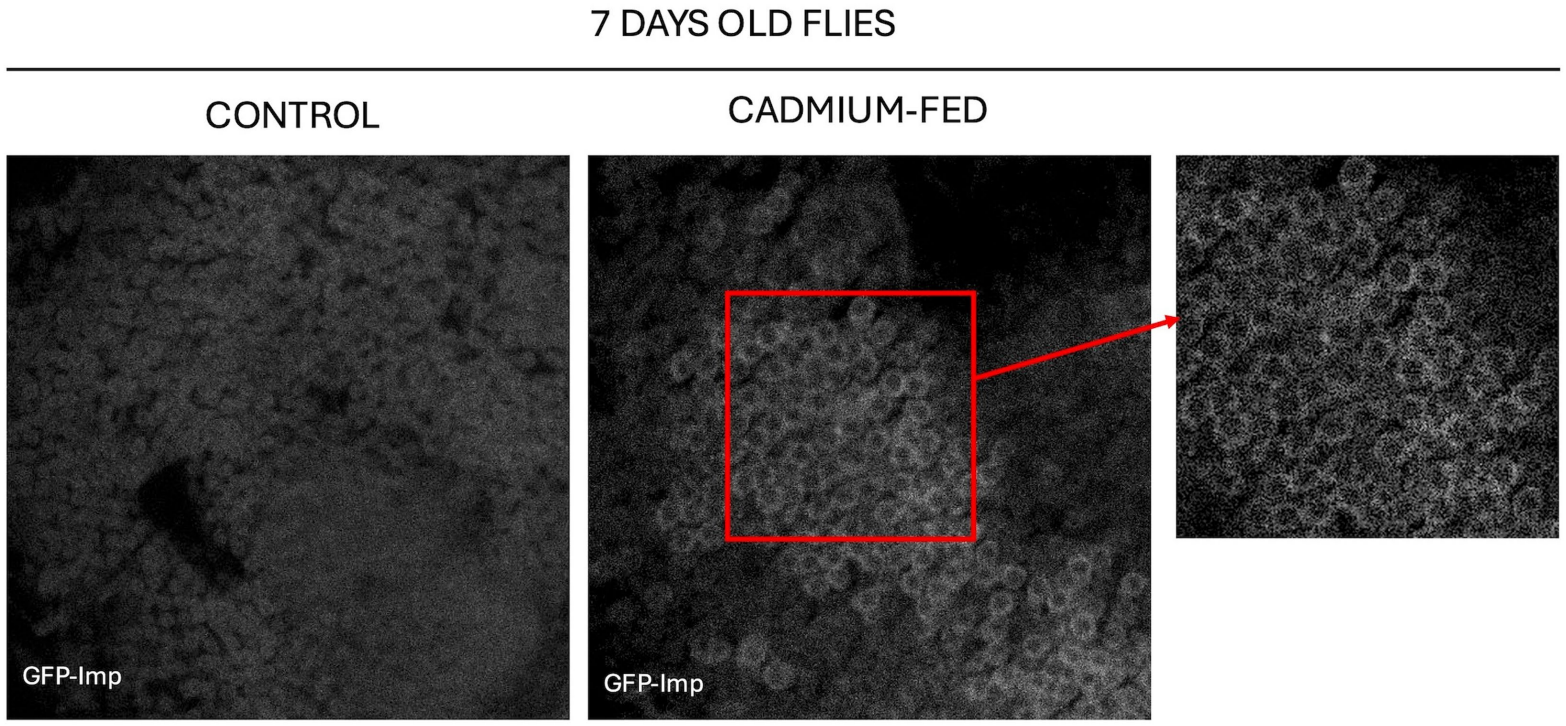
Confocal analysis of 7 days-old brains of *Drosophila melanogaster Imp-EGFP* untreated **(left)** and *Imp-EGFP* cadmium-treated plus magnified section **(right)**. In this strain Imp protein is tagged with GFP and brains are stained with anti-GFP (Novus, NB600-308). Magnification 63X.

To analyze the RNPs organization in neurons of cadmium-treated flies, we have observed the behavior of the RNA binding protein Imp. In *Drosophila* young brains, Imp accumulates into numerous small punctate structures and an increased condensation of Imp into large granules is observed in aged brains (Pushpalatha et al., 2022). We analyzed the level of Imp protein condensation in the brains of 7 days-old *Drosophila melanogaster Imp-EGFP* strain treated with cadmium, comparing them to untreated *Imp-EGFP* strain (Figure 9).

We observed that Imp protein accumulates into condensates in the brains of cadmium treated flies as early as 7 days of age, compared to the control.

## 4 Discussion and conclusion

Cadmium is known to disrupt cellular functions and induce neurotoxicity in different organisms and through various pathways. Understanding the molecular mechanisms by which cadmium affects neurons can help to identify potential therapeutic targets to counteract cadmium-induced neurotoxicity. In the *Drosophila* brain, we highlighted that cadmium affects cellular stress responses inducing neurodegeneration to the point of determining the alteration of complex neurological phenotypes.

With the aim of clarifying the molecular mechanisms underlying cadmium-induced neurodegeneration, we focused on the involvement of cadmium in influencing liquid–liquid phase separation (LLPS) in neurons. LLPS is a mechanism that enables RNA-binding proteins and RNAs to dynamically condense into membrane-less droplets. LLPS can be influenced by ionic concentrations within the cytoplasm. The ionic environment can affect the interactions between biomolecules, including proteins and RNA, thereby modulating their ability to undergo phase separation. High concentrations of certain ions can stabilize or destabilize interactions that lead to LLPS, ultimately affecting the formation of membrane-less organelles and condensates. For instance, research has shown that changes in ionic strength can impact the phase behavior of proteins involved in LLPS (Patel et al., 2017).

Among protein components of RNPs in *Drosophila* neurons, we focused on Imp to analyze its localization under cadmium stress conditions. Imp is a conserved RNA-associated protein known to localize to RNP granules in vertebrate and invertebrate neurons. In *Drosophila*, RNP granules containing Imp have been identified in the soma of Mushroom Body *γ* neurons, a crucial neuronal population for learning and memory processes (Vijayakumar et al., 2019; Formicola et al., 2021). We observed that in young brains subjected to cadmium treatment, the Imp protein accumulates in condensates more than in the control and as expected for old brains.

Our mechanistic hypothesis is that cadmium influences cytoplasmic ionic concentration, leading to an early or altered formation of condensates through liquid–liquid phase separation, triggering the activation of stress-related pathways that result in apoptosis and neurodegeneration.

In addition, recent findings suggest that phase separation is involved in the formation of chromatin domains, which can influence the accessibility of transcriptional machinery to specific genes, thereby regulating gene expression. Moreover, the interaction between liquid–liquid phase separation (LLPS) and chromatin structure is evident in the formation of heterochromatin (Strom et al., 2017). Our research opens the possibility that cadmium may also influence chromatin structure indirectly, contributing to neurodegeneration. In fact, the integrity and function of heterochromatin are critical for maintaining neuronal health, and disruptions in these processes are closely linked to the mechanisms underlying neurodegeneration (Napoletano et al., 2021).

This work requires further investigation, particularly into human cellular models, but it suggests that targeting the proteins involved in RNPs or using molecules that negatively influence LLPS could provide a pathway for targeted interventions to counteract the neurotoxic effects of cadmium.

## Data Availability

The original contributions presented in the study are included in the article/Supplementary material, further inquiries can be directed to the corresponding author/s.
