# Supplementary material for: Mechanistic insights into cadmium-related premature aging in Drosophila model
Source: Front Neurosci. 2025 Jun 4;19:1605687. doi: 10.3389/fnins.2025.1605687 (PMC12174419; doi:10.3389/fnins.2025.1605687)
Supplement: Supplementary file 1 [file Table_1.docx]

**Table S1.** Primer Sequences

| *rp49 Forward* | 5’ ATC GGT TAC GGA TCG AAC AA 3’ |
| --- | --- |
| *rp49 Reverse* | 5’ GAC AAT CTC CTT GCG CTT CT 3’ |
| *Toll-9 Forward* | 5’ ATC GGA TGA TGG GAA CAG TTG T 3’ |
| *Toll-9 Reverse* | 5’ GTA TTT CTT TGT GCT GTC CCT GA 3’ |
| *Thor Forward* | 5’ CTC CTG GAG GCA CCA AAC TTA TC 3 |
| *Thor Reverse* | 5’ TTC CCC TCA GCA AGC AAC TG 3’ |
| *p38b Forward* | 5’ TGA TGG ACG CCG ATC TGA AC 3’ |
| *p38b Reverse* | 5’ ATG CGA AGC TCA CAG TCC TC 3’ |
| *GstE5 Forward* | 5’ TTT GTG GAG ACC TTC CTC GC 3’ |
| *GstE5 Reverse* | 5’ AAA CGC CTG ACC CAT TCG AT 3’ |
| *GstD2 Forward* | 5’ AAG GAT GAC TAT CTG TTG CCC A 3’ |
| *GstD2 Reverse* | 5’ CAA ACG CGG TTT CGA TTC TCT 3’ |
| *Cyp4p3 Forward* | 5’ TGG CTC GTG GGA GCT TTT ATT 3’ |
| *Cyp4p3 Reverse* | 5’ GCC CTT CAC TAA TGG AGC AAT G 3’ |
| *TotM Forward* | 5’ CGA CAG CCT GGT CAC TTT CT 3’ |
| *TotM Reverse* | 5’ TAG CTT CAC CAG TGG CAA CC 3’ |
