# Supplementary figures and images for: Mechanistic insights into cadmium-related premature aging in Drosophila model
Source: Front Neurosci. 2025 Jun 4;19:1605687. doi: 10.3389/fnins.2025.1605687 (PMC12174419; doi:10.3389/fnins.2025.1605687)

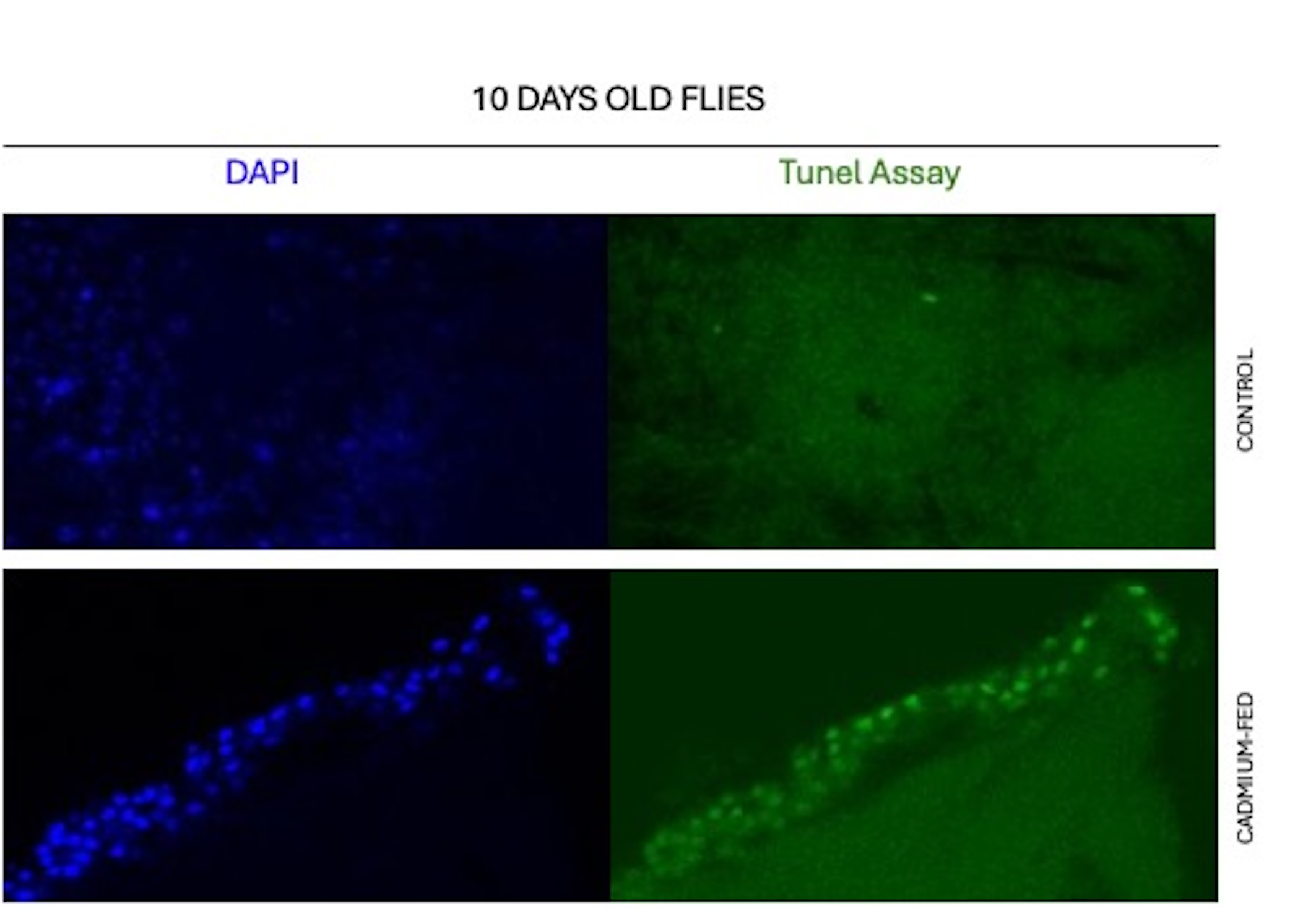

Supplement: Supplementary file 2 [file Image_1.jpeg]
